# The progress in predictive modeling of post-stroke epilepsy

**DOI:** 10.3389/fneur.2026.1877573

**Published:** 2026-07-09

**Authors:** Hao Chen, Lei Ge

**Affiliations:** Center for Rehabilitation Medicine, Rehabilitation & Sports Medicine Research Institute of Zhejiang Province, Department of Rehabilitation Medicine, Zhejiang Provincial People’s Hospital (Affiliated People’s Hospital), Hangzhou Medical College, Hangzhou, Zhejiang, China

**Keywords:** assessment, machine learning, post-stroke epilepsy, risk prediction models, stroke

## Abstract

Post-stroke epilepsy (PSE) is a significant complication of both ischemic (IS) and hemorrhagic strokes (HS), leading to increased morbidity and reduced quality of life. Accurate prediction of PSE risk is essential for early intervention and tailored management. Multiple predictive models have been developed for different stroke subtypes. In HS, models such as CAVE, CAVS, CAV+, and CAVE2 emphasize lesion characteristics and early seizures. Within IS, models such as SeLECT and PSEiCARe focus on cortical involvement, large-artery atherosclerosis, and early seizure occurrence. Recent advances in machine learning-based approaches have shown improved predictive accuracy for both IS and HS patients, although further validation is required for routine clinical application. This review summarizes and compares predictive models for PSE across stroke subtypes, highlighting their clinical relevance and potential for improving patient outcomes through early risk stratification. Integration of multimodal data may further enhance seizure prediction and guide personalized intervention strategies.

## 1 Introduction

Stroke is a major global health burden and remains the second leading cause of death and the third leading cause of disability worldwide (1). This neurological emergency is characterized by focal or global neurological dysfunction with high incidence, elevated disability rates, and substantial mortality (2). Stroke is pathologically classified into ischemic stroke (IS) and hemorrhagic stroke (HS) (3–5). Despite significant therapeutic advances, post-stroke sequelae remain critical determinants of patient outcomes (6). Among these complications, post-stroke epilepsy (PSE) affects 2–14% of the IS survivors and 10–20% following the HS (7–9). PSE is defined as recurrent unprovoked seizures following stroke in individuals without a prior history of epilepsy, with electrophysiological findings consistent with the lesion location (10). The condition contributes to increased morbidity, mortality, and reduced quality of life. Despite advances in acute stroke management, post-stroke epilepsy (PSE) remains a significant long-term complication, leading to recurrent seizures, increased morbidity, cognitive decline, and reduced quality of life. It is highlighting the need for early identification and prevention strategies (11).

According to the 2014 International League Against Epilepsy (ILAE) operational definition, seizures occurring within 7 days of a stroke are classified as acute symptomatic, whereas those occurring later are considered unprovoked (12). Acute symptomatic seizures result from transient stroke-related factors, while unprovoked seizures indicate a persistent epileptogenic predisposition and are used to define post-stroke epilepsy when recurrence risk is high (13). The incidence of ES ranges from 2 to 20%, while LS occurs in 6 to 15% of patients (14). ES predominantly manifests as generalized tonic–clonic seizures or focal seizures with impaired awareness, most commonly occurring within 24 h post-stroke (15). Associated neurological dysfunction in the lesion area causes altered consciousness, including perceptual, cognitive, and memory impairments. Clinical presentations include upward gaze deviation, laryngeal spasm, trismus, sustained muscle contractions, limb convulsions, and disturbance of consciousness with post-ictal amnesia. The LS primarily presents as focal seizures, often without consciousness impairment, characterized by brief (<1 min) unilateral limb or facial convulsions with abrupt onset and termination (16). When consciousness is impaired, patients exhibit sudden collapse, upward deviation of the eyes, and unresponsiveness. Automatisms may occur, including lip-licking, lip-smacking, chewing, swallowing, fumbling, aimless ambulation, and verbalization, with post-ictal amnesia. Enhanced cortical activity in the lesion area may facilitate focal to bilateral tonic–clonic seizures over time (17).

Machine learning (ML) is a computational discipline that models human learning processes through algorithmic approaches, constituting a pivotal subset of artificial intelligence primarily designed for structured data analysis (18–20). ML, an emerging interdisciplinary field, has been extensively applied in clinical medicine (21). AI-assisted stroke diagnosis has made significant advances in stroke lesion segmentation and classification, stroke risk prediction, and stroke prognosis (22). Recent studies suggest that ML-based models can improve the prediction of post-stroke seizures by integrating complex clinical and imaging data, enabling more accurate risk stratification and timely intervention (23). This technological advancement optimizes healthcare resource allocation, facilitates early identification of high-risk patients, and contributes to reducing stroke-related morbidity and mortality, demonstrating substantial clinical significance. The utilization of AI-driven applications has the potential to significantly reduce mortality rates and assist physicians in making informed treatment decisions. However, several important gaps persist in current research. Most existing predictive models are developed for single stroke subtypes and lack cross-subtype generalizability (24). In addition, models tailored to specific populations, particularly in China, remain limited. Although the ML approaches have demonstrated promising predictive performance, their clinical translatability is often constrained by model complexity, limited external validation, and poor integration into routine clinical workflows.

This review synthesizes current knowledge on risk factors for PSE and recent advances in machine learning-based prediction models for stroke outcomes, exploring effective assessment and therapeutic strategies to improve neurological and cognitive function in PSE patients.

## 2 Risk factors for post-stroke epilepsy

**Figure 1:**
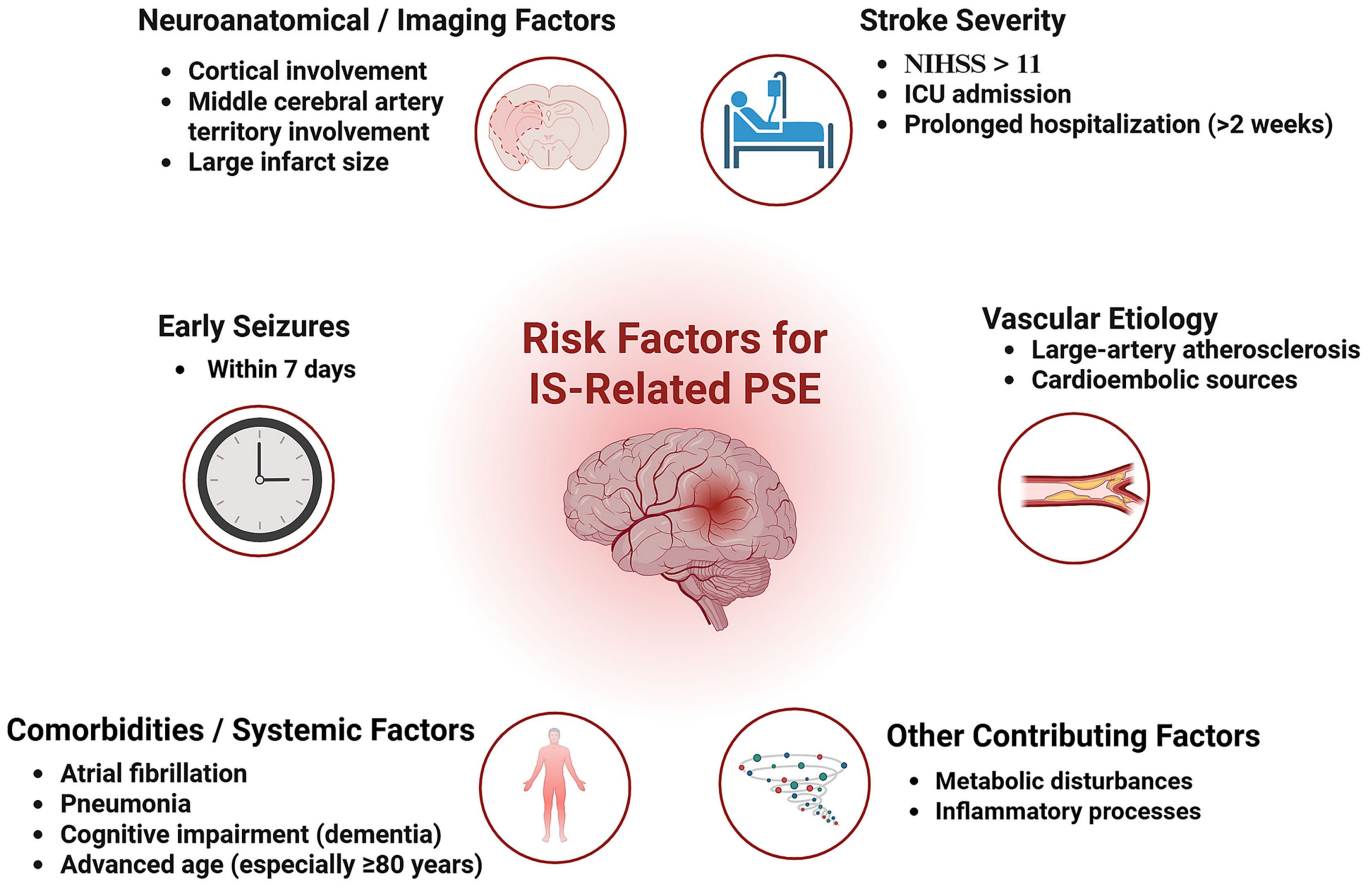
Comprehensive overview of the risk factors for IS-related PSE.

**Figure 2:**
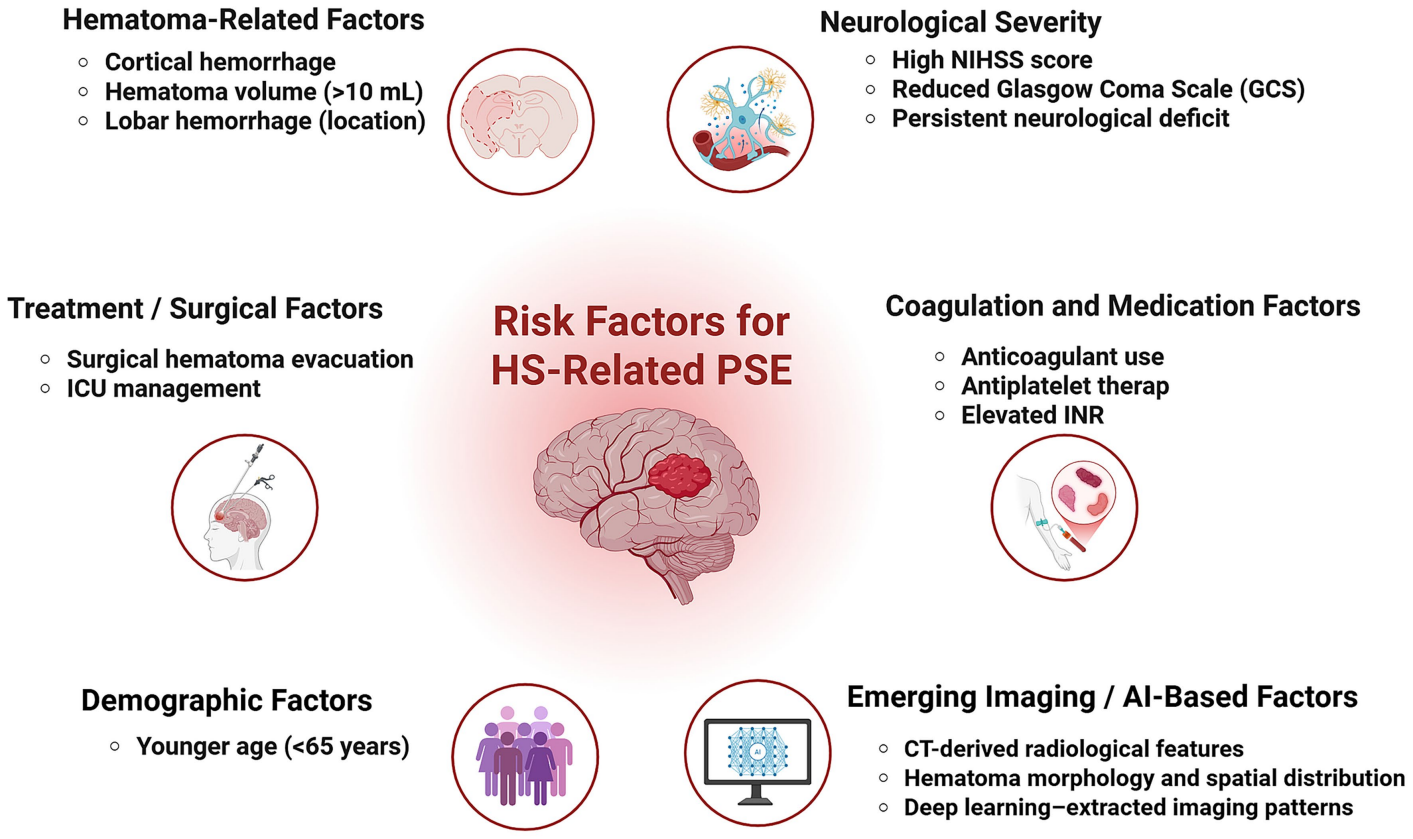
Comprehensive overview of the risk factors for HS-related PSE.

Understanding the risk factors associated with PSE is essential for the timely identification of high-risk patients and the development of effective predictive models. These risk factors vary depending on stroke type, lesion characteristics, patient demographics, and neurological status at onset (25, 26). In this section, we provide a detailed overview of the key determinants consistently associated with PSE development. The risk factors for PSE differ substantially between ischemic (Figure 1) and hemorrhagic stroke (Figure 2).

### 2.1 Age

Age is a widely recognized risk factor for PSE, with distinct patterns observed across stroke subtypes (27, 28). Multiple studies investigating HS have consistently identified younger age as an independent risk factor for PSE, with ages below 65 years serving as a critical threshold in predictive models (29). The underlying mechanism involves heightened inflammatory responses in younger patients following hemorrhage, resulting in increased neuronal excitability and subsequent seizure susceptibility. Moreover, seizure incidence following IS shows a U-shaped distribution across age groups, with a PSE incidence of 10.7% in patients younger than 65 years and 1.6% in those older than 85 years. The IS accounts for 30–50% of unprovoked seizures in patients aged ≥60 years, representing the most common etiology of epilepsy in this demographic (30). In elderly patients, the convergence of chronic comorbidities, declining cerebral function, and stroke-induced metabolic disruption creates a vulnerability cascade that increases seizure risk and mortality rates 2–3 fold compared to younger populations (31).

Following an initial post-IS seizure, the risk of recurrence reaches 90% within 6 to 12 months (32). In perinatal populations, acute ES following perinatal IS carries a 3-fold increased risk compared to late-onset seizures, with peak incidence occurring within the first year. Over 50% of pediatric patients develop active epilepsy within 10 years, and progressive neurological dysfunction in these patients may further evolve into drug-resistant epilepsy (33).

Age-related variations in PSE susceptibility necessitate individualized risk assessment, as seizure probability differs significantly between age groups and stroke subtypes, requiring comprehensive clinical evaluation for optimal management strategies.

### 2.2 Timing of first seizure onset

The timing of initial seizure onset represents a critical determinant of subsequent epileptic activity and long-term prognosis. Late-onset seizure has a peak within 6 to 12 months after the stroke and has a higher recurrence rate of up to 90% in both IS and HS (34). Late poststroke seizures tend to progress to poststroke epilepsy, with a recurrence rate of 71.5% within 10 years, significantly exceeding the 33% recurrence rate observed with ES (25).

In elderly populations with IS, LS predominate and exhibit higher recurrence rates than ES, potentially attributable to cortical hemosiderin deposition, neuronal reorganization, and functional alterations in peri-infarct tissue (35). EEG findings during the acute phase of stroke can potentially stratify the risk of subsequent seizures and predict the development of post-stroke epileptogenesis. Acute symptomatic seizures (*p* < 0.001) and epileptiform EEG abnormalities (*p* = 0.02) serve as robust predictors of PSE development, potentially associated with ipsilateral hippocampal sclerosis (36). A cohort subanalysis of 167 patients with acute seizures identified critical risk factors for PSE development: NIHSS score >14 (HR 2.98, 95% CI 1.57–5.67), longer interval from stroke to acute symptomatic seizures (days 4–7 post-stroke) (HR 2.51, 95% CI 1.37–4.59) and multiple acute symptomatic seizures (HR: 5.08, 95% CI: 2.58–9.99) were independently associated with PSE development (37).

The development of epilepsy, or recurrent seizures, after stroke occurs at varying rates, but it is higher after late-onset seizures than after early-onset seizures (34). Literature reports indicate that patients experiencing initial LS have a > 90% probability of subsequent seizure recurrence. ES also constitutes a risk factor for hemorrhagic stroke epilepsy by increasing metabolic demands in hypoxic tissue, promoting glial scar formation around lesion sites, causing long-term cerebral damage, and elevating LS incidence (38).

### 2.3 Lesion location in stroke

In the IS, the location of cerebral injury significantly influences the risk of PSE. Cortical involvement, in particular, is a well-established risk factor for epileptogenesis and varies across age groups. Studies have demonstrated that elderly patients (≥60 years) with cortical ischemia are more prone to generalized seizures. Imaging data from 60 elderly patients with IS revealed frequent involvement of the enlarged ventricles (83.3% ± 34.0%), subcortical basal ganglia (76.7% ± 37.0%), right hemisphere or temporal lobe (60.0% ± 37.9%), subcortical regions (56.7% ± 37.3%), frontal lobe (50.0% ± 35.4%), left hemisphere or diffuse cortical atrophy (43.3% ± 32.6%), temporal lobe (20.0% ± 17.9%), and occipital lobe (16.7% ± 15.2%). These findings suggest a strong association between large anterior circulation infarcts and a high incidence of seizures (39).

Seizures can further exacerbate neurological impairment following IS. Kumral et al. classified 76 patients with PSE into two groups based on neurological status within 48 h of seizure onset: transient neurological worsening (TNW) and long-lasting worsening (LLW) (40). A one-month follow-up with MRI-DWI revealed that LLW patients exhibited a higher frequency of multifocal lesions. This suggests that involvement of multiple lobes may lead to recurrent epileptic discharges, secondary infarct expansion, and hemorrhagic transformation. Emerging evidence also points to the role of enlarged perivascular spaces (EPVS) in the basal ganglia as predictors of recurrent IS (45.5% vs. 32.8%). Moreover, asymmetric distribution of EPVS in the centrum semiovale has been identified as an independent risk factor for PSE (OR = 3.7, 95% CI: 1.5–9.1), potentially related to small-vessel pathology, impaired cerebrospinal fluid drainage, electrophysiological imbalance, and disruption of the blood–CSF barrier (41).

According to the ICD-10 classification, ICH can be categorized into lobar hemorrhage (frontal, temporal, parietal, occipital), deep hemorrhage (basal ganglia, thalamus), brainstem, cerebellar, intraventricular, and mixed types (41). Cortical involvement is an independent risk factor for seizures following HS (26, 42). The incidence of seizures is particularly high in lobar hemorrhage due to its proximity to or direct involvement of cortical structures. Given the overlap in definition, lobar hemorrhage and cortical involvement are often analyzed together. Moreover, although hypertension is a major risk factor for deep ICH, it may paradoxically exert a protective effect against seizures. This is likely due to hypertensive hemorrhages occurring predominantly in deep regions such as the basal ganglia, sparing the cortex. This finding reinforces the pivotal role of cortical involvement in predicting post-ICH seizures (43).

### 2.4 Severity of stroke injury

The extent of stroke-induced brain injury is a critical factor in predicting the risk of PSE (44). According to the National Institutes of Health Stroke Scale (NIHSS), stroke severity is categorized as mild (≤3), moderate (4–10), or severe (>11). Severe IS has been identified as an independent risk factor for acute symptomatic seizures and the subsequent development of PSE (45). The timing of seizure onset is closely associated with moderate-to-severe strokes, cortical infarction, and large artery atherosclerosis (46, 47). Underlying mechanisms likely involve sustained excitotoxicity, neuroinflammatory cascades, and disruption of the blood–brain barrier, which together facilitate the formation and spread of epileptogenic networks, worsening neurological outcomes.

To improve the predictive accuracy of PSE, Redfors et al. integrated NIHSS scores with the TOAST classification and OCSP clinical subtypes, demonstrating that patients with total or partial anterior circulation infarctions exhibit greater stroke severity and are independently associated with PSE risk (45). In addition, a study assessing neurological impairment showed a positive correlation between the NIHSS score and PSE incidence, indicating its value in risk stratification and prognosis (48). In terms of treatment, a study of 153 patients with hyperacute IS who received intravenous alteplase revealed that 21 patients (13.7%) developed seizures (OR = 3.07, 95% CI: 1.22–7.75, *p* = 0.018), with 15 (9.8%) progressing to epilepsy. The mean initial NIHSS score in this group was 10.95 ± 6.25, decreasing to 2.09 ± 3.55 at 3 months. These findings suggest that higher NIHSS scores are associated with increased risks of seizures, hemorrhagic transformation, and poor neurological outcomes within 90 days post-thrombolysis, supporting its role as a predictor of adverse prognosis in PSE (49).

In HS, hematoma volume is also a key determinant of seizure risk (43, 50). Although definitions of significant hematoma volume vary across studies, most studies a volume >10 mL as a strong risk factor for post-hemorrhagic seizures (50). Larger hematomas exert greater mass effects, leading to more extensive neuronal injury and a higher likelihood of surgical intervention. Interestingly, some studies propose that smaller hematomas are more epileptogenic, particularly when located in cortical regions, possibly due to vascular anatomy. This highlights the central role of cortical involvement in the pathogenesis of PSE, a finding consistent across both IS and HS subtypes (51).

### 2.5 Other

Beyond conventional predictors, recent studies have identified several additional variables associated with PSE, particularly following HS. A meta-analysis revealed that chronic alcohol consumption is a significant risk factor, likely due to ethanol metabolites that enhance neuronal excitability and lower seizure threshold. Elevated NIHSS scores at admission and comorbid coronary artery disease have also been associated with increased risk of PSE (52). Emerging evidence has highlighted the role of metabolic and inflammatory biomarkers in seizure prediction. Hypercholesterolemia has been shown to have an inverse association with ICH, and statin therapy appears to reduce ICH risk without increasing seizure incidence (53). Notably, lower levels of tumor necrosis factor receptor 1 (TNF-R1) and elevated levels of neural cell adhesion molecule (NCAM) were identified as independent blood-based predictors of PSE (54). Models that combine these biomarkers with clinical variables demonstrate improved predictive performance compared with models that use single predictors.

Surgical intervention—particularly hematoma evacuation—has also been implicated as a potential trigger for seizures (51). Nonconvulsive status epilepticus (NCSE), though often underdiagnosed, is increasingly recognized in HS patients and has been independently linked to temporal lobe involvement and surgical treatment (55). Comorbid conditions and pharmacotherapy are additional factors influencing PSE outcomes. Most patients with IS-related epilepsy exhibit at least two conventional risk factors, with hypertension, diabetes mellitus, dyslipidemia, and a history of smoking or alcohol use being most prominent. These variables not only increase seizure susceptibility but are also critical targets for primary and secondary stroke prevention. Furthermore, diabetes (OR = 5.242, *p* = 0.004), elevated homocysteine levels (OR = 1.103, *p* = 0.033), and polytherapy with antiseizure medications (OR = 3.354, *p* = 0.019) have been associated with cognitive impairment in post-stroke patients (56).

Among adult males with cortical IS and comorbidities such as hypertension, hyperlipidemia, or coronary artery disease, the frequency of seizure recurrence is notably higher (57). Stroke-related complications and polypharmacy further increase mortality risk. For instance, post-stroke depression affects 25–79% of patients and is frequently treated with selective serotonin reuptake inhibitors (SSRIs). A nationwide cohort study reported a 2.74-fold increased risk of seizures in IS patients using SSRIs compared to non-users, possibly due to drug-induced hyponatremia and altered neuronal ionic homeostasis (58). In high-risk patients, the selection of antiseizure medications must account for pharmacokinetic and pharmacodynamic interactions. Common enzyme-inducing drugs such as carbamazepine and phenytoin may reduce plasma concentrations of anticoagulants or antihypertensives, compromising control of comorbidities. Conversely, switching to non–non-enzyme-inducing agents may increase serum drug levels and toxicity, thereby complicating epilepsy management post-stroke (30).

Moreover, a study using NHANES data (1999–2006 and 2011–2018; *n* = 30,443) developed an XGBoost model to estimate trunk fat percentage from five anthropometric variables (sex, waist circumference, height, weight, age), achieving an *R*^2^ of 0.8450 on the test set (99.3% of the full model’s accuracy). External validation in the CHARLS cohort (*n* = 13,524) demonstrated that the estimated trunk fat percentage consistently outperformed whole-body fat percentage in discriminating cardiometabolic conditions, with particular prominence for stroke, alongside hypertension, dyslipidemia, diabetes, and heart disease. The average relative improvement in AUC across all endpoints was 2.77% (e.g., diabetes: 3.22%) (59). Thus, trunk fat percentage derived from simple anthropometric measurements using machine learning serves as a superior non-invasive biomarker of central adiposity, especially for stroke risk assessment.

### 2.6 Stroke subtypes

The risk of post-stroke epilepsy (PSE) varies significantly across different stroke subtypes. HS comprises intracerebral hemorrhage (ICH) and subarachnoid hemorrhage (SAH), accounting for 15.0–30.0% of all stroke cases, with mortality rates four times higher than the IS (4, 5).

In IS, seizures are primarily associated with cortical involvement, large infarct size, and severe neurological deficits (60). Although the overall incidence of PSE is lower than in hemorrhagic stroke, IS contributes substantially to the total burden of PSE due to its higher prevalence. Moreover, the risk of seizures is generally higher, particularly in patients with lobar hemorrhage, larger hematoma volume, and cortical extension in ICH. The presence of early seizures is also a strong predictor of subsequent epilepsy in this subgroup (61). In SAH, seizure occurrence is often related to aneurysmal rupture, intracerebral extension, and secondary complications such as cerebral vasospasm. Early seizures may result from acute irritation caused by subarachnoid blood, whereas delayed seizures are more likely associated with secondary ischemic injury and gliosis.

Overall, hemorrhagic stroke subtypes (ICH and SAH) tend to carry a higher risk of both early and late seizures compared to IS, likely due to more pronounced cortical irritation and acute structural damage. Understanding these differences is important for risk stratification and tailored clinical management.

## 3 Predictive models for post-ischemic stroke epilepsy

In recent years, researchers have developed several predictive models for PSE in IS patients based on established risk factors. Most existing models use scoring systems as screening tools, which offer high clinical feasibility due to their simplicity and ease of use. These models enable early identification of high-risk individuals, which can inform timely and personalized interventions. By reducing seizure incidence in vulnerable populations, such tools play a crucial role in improving patient prognosis, guiding initiation of antiseizure therapy, and mitigating seizure burden in the post-stroke period (Table 1).

**Table 1:** Comparison of prediction models for post-stroke epilepsy.

Model	Target population	AUC (Derivation/Validation)	Sensitivity	Specificity	NPV	Prediction timeframe	Scoring simplicity	Clinical usability	Advantages	Limitations
SeLECT	Mild ischemic stroke	0.77/~0.75	Moderate (~70%)	High	High	Late-onset (months–1 year)	High	High	Simple and fast screening tool; Suitable for ruling out late-onset PSE	Lacks coverage of comorbidities, medication history, and genetic risk factors
PSEiCARe	Elderly ischemic stroke patients	0.762/0.792	Moderate	Moderate	Moderate	Within 1 year	Moderate	Moderate	Risk stratification for elderly patients; Real-world clinical validation	No EEG or imaging input; Narrower generalizability
PoSERS	Ischemic and hemorrhagic stroke patients	-	70%	99.6%	98.8%	Early and late-onset	Low	Moderate	Broad applicability; High specificity; Considers stroke location and type	Complex scoring system; Requires complete imaging and clinical datasets
Machine learning	High-risk stroke, neurocritical patients	~0.93	91.88%	86.13%	N/A	Real-time/continuous	Low	Low–Moderate	High accuracy (up to 93.40%); Enables early pharmacologic intervention	High technical demand; Dependent on EEG quality and deep learning model robustness

### 3.1 SeLECT scoring system

The SeLECT score is one of the most widely used and specific predictive models for PSE following IS. It incorporates five major risk factors: Severity of stroke (S), Large-artery atherosclerosis (L), Early seizures (E), Cortical involvement (C), and involvement of the Territory of the middle cerebral artery (T). It was derived from a Swiss cohort of 1,200 first-ever ischemic stroke patients and externally validated in three independent cohorts from Austria, Germany, and Italy, totaling 1,169 participants. Each factor is assigned points to generate a total score ranging from 0 to 9, it provides individualized 1- and 5-year risk estimates (0.7–63% and 1.3–83%, respectively) with good discrimination (AUC = 0.77) and calibration. This tool demonstrates a high negative predictive value, making it particularly useful for ruling out PSE in patients with mild IS. It is considered an effective predictor of late-onset seizures (62).

However, the model has notable limitations. It does not fully account for several key factors that may influence PSE risk, including genetic predispositions, pre-existing comorbidities (e.g., hypertension, diabetes, depression), medication history, neuroimaging-based assessments of injury severity, and potential biases in follow-up due to patient self-reporting. As such, its positive predictive value remains relatively low, and its outcomes should be interpreted in conjunction with broader clinical evaluations (63). The Scandinavian Stroke Scale (SSS), though not a dedicated predictive tool, can serve as an initial screening measure for stroke severity and be used in combination with the SeLECT score to enhance overall stratification.

### 3.2 PSEiCARe scoring system

The PSEiCARe (Post-Stroke Epilepsy in the Elderly – Ischemic Stroke Cohort Assessment and Risk Evaluation) score was developed using a large population-based Taiwanese National Health Insurance claims cohort of ischemic stroke patients from 2003 to 2014. A total of 125,757 patients were included, with 87,068 assigned to the development cohort and 38,689 to the temporal validation cohort. It aims to assess the risk of late-onset PSE within 1 year of the initial event, which shows acceptable discrimination in both the development and validation cohorts (0.762 and 0.792, respectively). The model includes seven clinical variables with a total score ranging from 0 to 16: prolonged hospitalization (>2 weeks, 1 point), seizure at admission (6 points), advanced age (>80 years, 1 point), intensive care unit (ICU) admission (3 points), cognitive impairment (2 points), atrial fibrillation (2 points), and pneumonia on admission (1 point). Based on the total score, patients are stratified into four risk categories: low (0), moderate (1–5), high (6–10), and very high (>11) (64). The study further identified several independent predictors of PSE, including advanced age (≥80 years), dementia, atrial fibrillation, pneumonia, hypertension, diabetes, dyslipidemia, and malignancy. Atrial fibrillation, in particular, is a common cause of cardioembolic stroke and is associated with cortical ischemic lesions, spontaneous arterial recanalization, and hemorrhagic transformation—factors that may increase PSE risk. Therefore, early identification and effective management of atrial fibrillation may help reduce the incidence of PSE. Notably, the SeLECT score showed superior predictive accuracy in patients with cerebral infarction.

### 3.3 PoSERS scoring system

The Post-Stroke Epilepsy Risk Scale (PoSERS) is a predictive model applicable to both IS and HS patients. Developed in 2010 by German researchers, the model was based on a prospective 1-year study involving 264 stroke patients, with 6- and 12-month follow-ups, integrating clinical and neuroimaging data to assess the risk of PSE (65). The PoSERS model includes 10 risk variables, such as stroke location (supratentorial vs. infratentorial), persistent neurological deficits (modified Rankin Scale [mRS] > 3), stroke subtypes (e.g., ICH with cortical involvement, IS with secondary hemorrhage), diagnosis of vascular encephalopathy, and the timing of seizures (early- or late-onset). Among the participants, 148 underwent electroencephalogram (EEG) evaluation. Seven key predictive factors were identified via chi-square analysis: supratentorial stroke, cortical hemorrhage, cortical or cortico-subcortical ischemia, IS with persistent neurological deficits, mRS > 3, seizures within 14 days of stroke, and seizures occurring 15 days or later post-stroke. The study reported early-onset seizures in 4.5%, at least one late seizure in 6.4%, and post-stroke epilepsy in 3.8% of patients within 1 year. The model demonstrated a high specificity (99.6%) and negative predictive value (98.8%), with a moderate sensitivity (70%) and a positive predictive value of 87.5% (65). Weighted analysis further highlighted supratentorial stroke, cortical hemorrhage, and late-onset seizures (≥15 days) as the most robust predictors. Despite its high specificity, the PoSERS score has limited sensitivity to EEG findings—possibly due to insufficient monitoring duration—and requires comprehensive clinical data and standardized stroke classification, making it relatively complex for routine use. Nevertheless, PoSERS may be particularly valuable for identifying patients at high risk of early-onset seizures after both IS and HS. It can inform early therapeutic decisions and guide individualized seizure prevention strategies. However, the study was limited by its small single-center sample and only moderate sensitivity, and EEG added limited prognostic value. Future validation with larger, multicenter cohorts and improved EEG integration is warranted to enhance its clinical applicability.

### 3.4 Machine learning-based prediction models

The rapid advancement of artificial intelligence has significantly enhanced medical diagnostic capabilities, particularly in the early prediction of PSE. Machine learning models integrated with electroencephalogram (EEG) monitoring have emerged as a promising approach to identify seizure onset. These models typically involve EEG signal acquisition, preprocessing, feature extraction, and classification of seizure states (66). Compared with conventional EEG-only monitoring, machine learning models have achieved a true positive rate of up to 92.23% for detecting preictal (pre-seizure) states. Early prediction enables timely pharmacological intervention, which is particularly critical for neurocritical care patients, such as those in status epilepticus. Recent approaches have incorporated long-term recurrent convolutional networks (LRCNs), which combine convolutional neural networks (CNNs) with long short-term memory (LSTM) blocks (67). These deep learning models automatically extract spatiotemporal features from EEG time-series data, creating robust frameworks for seizure prediction in post-stroke patients.

In EEG segment-based evaluations, such models have demonstrated an accuracy of 93.40%, a sensitivity of 91.88%, and a specificity of 86.13%. Compared to traditional machine learning methods, these represent a 5–9% improvement in both sensitivity and specificity. The enhanced predictive performance facilitates earlier clinical decision-making and more effective seizure prevention, underscoring the potential of AI-assisted EEG analysis in stroke-related epilepsy management.

## 4 Predictive models for post-hemorrhagic stroke epilepsy

Clinical prediction models use existing clinical data to develop statistical tools that estimate the probability of specific outcomes in defined medical contexts. Their core principle lies in inferring unknown outcomes based on known variables. In the context of PSE, various predictive models have been developed both domestically and internationally, targeting different risk factors. Commonly used models include the PoSERS model, the CAVE score, and models incorporating electroencephalographic monitoring combined with machine learning or deep.

learning algorithms. Each model presents unique characteristics and applications (Table 2), providing valuable support for the early identification and intervention of PSE.

**Table 2:** Comparison of predictive models for post-ICH epilepsy.

Model name	Year/Region	Included factors	Score range	Primary outcome	C-statistic (Derivation/Validation)	Sensitivity (%)	Specificity (%)	Advantages	Limitations
CAVE	2014 Europe	Cortical involvement; Age <65; Hematoma >10 ml; Early seizure	0–4	LS	0.81/0.69	15–35% (high-risk subset, scores 3–4)	—	Simple to calculate; Validated in prospective cohort	Small sample size; No EEG data; Underestimated LS incidence
CAVS	2020 USA	Cortical hemorrhage; Younger age; Larger hematoma; Surgical evacuation	0–4	LS	0.76	—	—	Validated across multi-ethnic large cohort; Practical use	Short follow-up, lack of seizure types and EEG data
LANE	2021 China	Lobar hemorrhage; Age <65; NIHSS >15; Early seizure	0–6	LS	0.83/0.78	59.6% (score >2)	87.7%	Tailored for Chinese population; High predictive accuracy	No biomarkers, neurophysiological or surgical data included
CAV+	2022 USA	CAV plus anticoagulant; Antiplatelet use; GCS, INR, SBP	—	ES	AUC 0.79 (Xgboost)	91.9%	86.1%	Machine learning enhances predictive performance	Limited sample; Requires multi-center validation
CAVE2	2023 Taiwan	Cortical involvement (2 pts); Age <65; Hematoma >10 ml; Early seizure	0–5	LS	0.74	—	—	Better high-risk discrimination; Refined risk stratification	Single-center, small sample; Lacked EEG and serial NIHSS

### 4.1 The CAVE model

The CAVE model, developed by European researchers in 2014, was based on a 6-year follow-up of 993 patients with HS (68). Using Kaplan–Meier analysis and log-rank tests, it assessed annual incidence rates of early-onset seizures (ES) and late-onset seizures (LS). The model incorporates four predictors: cortical involvement, age <65 years, hematoma volume >10 mL, and ES, each assigned 1 point, yielding a total score ranging from 0 to 4. Corresponding 5-year seizure risks for CAVE scores of 0 to 4 were 0.6, 3.6, 9.8, 34.8, and 46.2%, respectively. Notably, patients with scores of 3–4 accounted for only 15%, indicating limited sensitivity in predicting future seizures. Primarily designed to predict late-onset seizures (i.e., >1 week post-HS), the CAVE model offers the advantages of simplicity and validation in an independent prospective ICH cohort. However, its discriminative performance varies: the C-statistic was 0.81 (95% CI: 0.76–0.86) in the derivation cohort but decreased to 0.69 (95% CI: 0.59–0.78) in the validation cohort, possibly due to limited sample size. Additionally, the model excludes neurophysiological data such as EEG, potentially overlooking subclinical seizures. Despite its extended follow-up, the model may underestimate the lifetime risk of LS following ICH. Thus, while the CAVE model is useful for individual risk stratification, it remains insufficient as a tool for clinical decision-making.

### 4.2 The CAVS model

The CAVS model was introduced in 2020, based on the ERICH (Ethnic/Racial Variations of Intracerebral Hemorrhage) study—a large prospective investigation aimed at examining racial disparities in epilepsy following ICH (26). A total of 3,000 patients were enrolled, including 1,000 non-Hispanic White, 1,000 non-Hispanic Black, and 1,000 Hispanic participants. Ultimately, 2,507 patients met the inclusion criteria. The study collected detailed demographic data, clinical history, pre-ICH medication and substance use, and information on acute hospitalization. Hematoma location and volume were determined via CT imaging, and survivors were followed up at 3, 6, and 12 months. In this cohort, ES did not reach statistical significance as an independent predictor. Thus, the CAVS model modified the original CAVE score by replacing ES with surgical hematoma evacuation, incorporating four variables: cortical involvement, younger age, larger hematoma volume, and surgical evacuation. Each factor contributed one point. For each unit increase in the CAVS score, the odds ratio for late-onset seizures (LS) increased by 2.8 (95% CI: 2.2–3.5, *p* < 0.0001). The C-statistic for the CAVS model was 0.76, compared to 0.73 for the CAVE model, though the difference was not statistically significant (AUC difference *p* = 0.0799).

The CAVS model validated the CAVE score’s applicability across multiethnic populations, showing no significant differences in seizure risk across racial groups. Furthermore, ES was not an independent risk factor for LS, whereas surgical hematoma evacuation was a significant predictor. Despite its strengths in cohort diversity, the CAVS study was limited by shorter follow-up duration and reliance on interview-based assessments, which may have underestimated LS incidence. Moreover, the lack of data on seizure types and neurophysiological findings constrained mechanistic insights.

Regarding antiseizure medication (ASM) use, the study suggested that prophylactic ASM administration may reduce ES incidence and future hospitalizations, potentially mitigating LS risk (69). However, due to multicollinearity, ASM use was not excluded from the analysis. Future randomized controlled trials are needed to clarify the benefits of ASM in high-risk individuals identified by the CAVS model.

### 4.3 The LANE model

The LANE model was developed in 2021 by a major stroke center in Qingdao, China, to improve prediction of late-onset seizures (LS) following HS in Chinese populations (42). Unlike the CAVE model, which was developed from European cohorts, the LANE model addresses the higher incidence of HS observed in East Asia, as highlighted by the Global Burden of Disease studies. A total of 602 patients were included in the study. Using multivariable Cox regression, four independent risk factors were identified: lobar hemorrhage and age <65 years (each assigned 1 point), NIHSS score >15, and ES (each assigned 2 points), yielding a total LANE score ranging from 0 to 6. In the derivation and validation cohorts (*n* = 521), the LANE model achieved C-statistics of 0.83 and 0.78, respectively, outperforming the CAVE model, which achieved C-statistics of 0.81 and 0.74 under the same conditions. When the LANE score exceeded 2, the model demonstrated a sensitivity of 59.6% and a specificity of 87.7% for predicting LS.

The LANE model represents the first LS prediction tool derived from a Chinese ICH population, offering strong discriminative ability and clinical relevance (70). However, its limitations include the exclusive use of routine clinical data without incorporating genetic markers, blood-based biomarkers, neurophysiological testing, or surgical intervention metrics. Future studies are warranted to investigate PSE risk factors across diverse regions, ethnicities, and environmental backgrounds within China, which may help refine and expand the predictive capacity of the LANE model.

### 4.4 The CAV+ model

With advances in technology, machine learning (ML) has increasingly been applied to medical data analysis and outcome prediction. In 2022, Bunney et al. developed the CAV+ model to predict ES following HS, building upon the original CAVE model (71). The study included 864 patients and introduced additional variables—namely, anticoagulant use, antiplatelet use, Glasgow Coma Scale (GCS), International Normalized Ratio (INR), and systolic blood pressure—to enhance the predictive capacity. Multiple machine learning algorithms were employed to construct and compare the CAV and CAV+ models, including logistic regression, LASSO regression, support vector machines (SVMs), gradient boosting (XGBoost), and random forests (RFs). Model performance was assessed using receiver operating characteristic (ROC) curves. In the XGBoost model, the CAV+ model achieved an AUC of 0.79 (95% CI: 0.71–0.87), significantly higher than the CAV model’s AUC of 0.72 (95% CI: 0.62–0.82, *p* = 0.04). Similarly, in the LASSO model, the CAV + AUC was 0.77 (95% CI: 0.68–0.85), compared to 0.69 for the CAV model (95% CI: 0.58–0.80, *p* = 0.02), indicating superior predictive performance. The XGBoost-based CAV+ model identified hematoma location (weight 0.69), GCS score (0.15), and INR (0.15) as the most influential predictors. These findings confirm that ES are indeed predictable using multivariable models and that extending the original CAVE framework with additional variables improves performance. Notably, the CAV+ model also incorporates electroencephalogram (EEG) data and insights, which may help optimize the use of prophylactic antiseizure medications (ASMs) (72).

Although still in the exploratory phase, the CAV+ model demonstrates strong potential and scalability. Further validation in larger, multi-center, and multi-ethnic cohorts is warranted to refine its clinical utility.

### 4.5 The CAVE2 model

The CAVE2 scoring system was developed in 2023 by researchers in Taiwan based on an analysis of 408 patients with ICH, aiming to refine the prediction of late-onset seizures (LS) (73). Given that cortical involvement is the strongest risk factor for seizures after ICH, the CAVE2 model adjusted the weight of this variable—assigning 2 points instead of 1—while retaining the original CAVE model’s other components, resulting in a total score ranging from 0 to 5. The study found that LS incidence under the CAVE2 scoring system was 4.6% for scores ≤1 (12/258), 18.3% for scores of 2 (13/71), and 54.4% for scores >3 (20/37). In comparison, the original CAVE model yielded LS risks of 6.7% (<1 point, 17/255), 14.8% (2 points, 17/115), and 47.4% (>3 points, 18/38). The C-statistics were 0.73 for CAVE and 0.74 for CAVE2, indicating comparable overall predictive performance. However, CAVE2 demonstrated enhanced risk discrimination for patients at the higher end of the score spectrum, with over a quarter of patients scoring >2 eventually developing LS. The study also highlighted that antiseizure medications (ASMs) may reduce the incidence of post-ICH seizures. Nevertheless, the CAVE2 model has several limitations. It was based on a single-center, small-sample study and did not incorporate potentially valuable markers such as blood-based indicators, electroencephalographic (EEG) data, or serial NIHSS scores. EEG was performed in only a minority of patients. Moreover, patients lost to follow-up within 1 year were excluded, which may have led to an underestimation of the 1-year seizure incidence.

In the future, more researches should aim to validate the CAVE2 model in larger, multi-center cohorts, integrating multimodal data to enhance predictive accuracy and clinical utility.

### 4.6 Others

Recent advances in artificial intelligence have introduced novel approaches for predicting PSE, particularly in patients with ICH. The new study developed a deep-learning model (PSENet) based on non-contrast computed tomography (NCCT) imaging to predict PSE risk after initial ICH (74). This study included 1,130 patients, among whom 62 developed PSE, and demonstrated that the model achieved superior predictive performance compared with conventional clinical scoring systems, with an AUC of 0.840 and an accuracy of 0.876. Notably, the model integrated imaging-derived features such as cortical involvement and hematoma volume, which are key factors in epileptogenesis, and outperformed traditional clinical models, including CAVE and LANE scores. These findings suggest that deep-learning–based imaging analysis can capture complex spatial and pathological patterns that are not fully reflected in conventional risk scores, providing a promising direction for improving individualized prediction of post-hemorrhagic PSE (75).

## 5 Conclusion

Post-stroke epilepsy (PSE) remains a clinically significant complication, with well-established risk factors including cortical involvement, hemorrhagic stroke, early seizures, and selected comorbidities. However, several important gaps persist: most existing predictive models focus on single stroke subtypes and lack cross-subtype generalizability; models tailored to specific populations—particularly in China—remain limited, and machine learning approaches, while promising, often face constraints in clinical translatability due to complexity, limited external validation, and poor integration into routine workflows. These limitations underscore the need for models that are both accurate and broadly applicable across diverse patient populations.

Future research should prioritize the development and external validation of multimodal prediction frameworks integrating clinical, imaging, and electrophysiological data in large, multicenter cohorts. Prospective and randomized studies are also needed to determine whether early identification of high-risk patients can inform effective preventive strategies, including timely antiepileptic therapy. Efforts to standardize prediction methodologies, improve model interpretability, and facilitate real-world clinical implementation will be essential for bridging the gap between predictive performance and the personalized management of PSE.
